# Squamous Cell Carcinoma: A Clinical and Histopathological Review of a South African Tertiary Dermatology Unit

**DOI:** 10.1155/jskc/8884436

**Published:** 2025-06-20

**Authors:** Ahmed Dadoo, Bianca Tod, Johann Schneider, Willem Visser

**Affiliations:** ^1^Division of Dermatology, Department of Medicine, Faculty of Medicine and Health Sciences, Stellenbosch University and Tygerberg Academic Hospital, Cape Town, South Africa; ^2^Division of Anatomical Pathology, Department of Pathology, Faculty of Medicine and Health Sciences, Tygerberg Academic Hospital, Stellenbosch University and National Health Laboratory Service, Cape Town, South Africa

## Abstract

**Background:** Cutaneous squamous cell carcinoma (CSCC) is the second most prevalent form of skin cancer globally. However, its incidence is rising relative to basal cell carcinoma (BCC) in countries such as Australia and the United States. In South Africa, where the population is exposed to numerous CSCC risk factors, including high ultraviolet radiation levels and a high burden of immunosuppression, there remains a notable paucity of scientific literature characterising the disease within this context.

**Aims:** To describe the clinical, histopathological and risk profile characteristics of CSCC in a South African cohort to identify patterns that can inform local clinical practice and guide future research priorities.

**Methods:** A retrospective data analysis of patients seen at Tygerberg Academic Hospital between 1 September 2019 and 31 August 2020 was conducted. Demographic and clinical data were extracted from medical records. Two study evaluators used predetermined criteria to review the histopathological features in skin biopsies. Risk stratification of lesions followed the guidelines of the National Comprehensive Cancer Network.

**Results:** Over one year, 113 CSCCs were diagnosed in 83 patients. Participants were primarily Fitzpatrick skin phototype I (65.1%) and male (60.2%), with a median age of 73 years and a male-to-female ratio of 1.51:1. The BCC-to-CSCC incidence ratio was 1.71:1. Most lesions had been present for over 6 months (87.6%) and were located on the head and neck (59.3%). Punch biopsies diagnosed 62.8% of lesions. Histologically, 63.7% were well differentiated, and 40.7% were invasive. Conventional CSCCs comprised 75.2% of lesions; 5.3% were the high-risk acantholytic subtype. A high recurrence risk was found in 77.0% of lesions.

**Conclusion:** This study highlights the heterogeneous nature of CSCC in South Africa and underscores the need for prospective, context-specific research to enhance prevention, early detection and management efforts nationwide.

## 1. Introduction

Cutaneous squamous cell carcinoma (CSCC) is the second most prevalent cutaneous malignancy globally, after basal cell carcinoma (BCC) [1–3]. Yet its incidence is rising relative to BCC in countries such as Australia and the United States [4–6]. While CSCC is often curable with early intervention, a subset of cases can progress to invasion, metastasis and death [7–10]. The South African population is exposed to multiple risk factors for CSCC, including consistently high year-round ultraviolet radiation (UVR), a high prevalence of HIV infection and oculocutaneous albinism (OCA) and widespread outdoor occupational and recreational exposure [11–17]. However, there remains a notable lack of scientific literature characterising CSCC in the South African setting. To address this gap, the present study aimed to characterise the clinical, histopathological and risk profile characteristics of South African patients who presented with CSCC at a tertiary dermatology unit in the Western Cape Province. By identifying patterns within this cohort, the study contributes to a more nuanced understanding of CSCC in South Africa, intending to inform future research and clinical practice.

## 2. Materials and Methods

### 2.1. Study Design

This retrospective descriptive study included patients who attended the Tygerberg Academic Hospital (TAH) Dermatology Outpatient Department (OPD) with histologically proven new or recurrent CSCC between 1 September 2019 and 31 August 2020. The recording of Fitzpatrick skin phototypes became mandatory as of 1 September 2019 at the TAH Dermatology OPD.

### 2.2. Study Setting

TAH is a publicly funded academic tertiary-level hospital in Cape Town, Western Cape Province, South Africa, serving a large catchment area that includes primary and secondary healthcare facilities. TAH serves a population with low- to middle-income status. The Dermatology OPD is staffed by nurses, dermatology registrars and consultant dermatologists. Minor procedures are performed daily. Confirmed high-risk or surgically challenging tumours are generally referred to the Division of Plastic and Reconstructive Surgery at TAH for further management.

### 2.3. Study Sample

One hundred and thirteen lesional biopsies confirmed CSCC in 83 patients during the 1-year study period. The study included patients aged 18 years or older with histologically confirmed CSCC. Patients with incomplete medical records were excluded from the study. Patients for whom histology slides of lesions could not be retrieved were also excluded. Lesions classified as keratoacanthomas were excluded after review by the investigators.

### 2.4. Data Collection

Patient data were obtained from electronic doctors' notes stored in the OpenText Enterprise Content Management system of TAH. Pathology reports were accessed through the National Health Laboratory Service (NHLS) following the Guidelines for Requests for Access to Biospecimens, Data and Resources for Research and Related Activities. Histology slides stored by the Division of Anatomical Pathology archive at the Tygerberg NHLS were retrieved to review histological features according to a predefined set of criteria by a dermatology registrar (A.D.) and an anatomical pathologist (J.W.S.). The principal investigator (A.D.) collated the anonymised data in a Microsoft Excel spreadsheet.

### 2.5. Patient Characteristics

The Fitzpatrick skin phototypes of patients were recorded in the clinical records. Patients were recorded as immunosuppressed if they were HIV positive, used immunosuppressive medication, were organ transplant recipients or had a haematological malignancy.

### 2.6. Diagnosis of CSCC

Registrars and consultants from the Division of Dermatology at TAH performed skin biopsies. In some cases, patients had more than one CSCC, requiring multiple biopsies at the same visit. Lesion risk stratification was performed according to National Comprehensive Cancer Network guidelines.

An anatomical pathologist (J.W.S.) and A.D. retrieved and reviewed histopathology sections of cases where the pathology reports did not include adequate information regarding diagnosis, histological growth pattern or perineural involvement. J.W.S. reviewed the CSCC specimens and categorised them according to the fourth edition of the World Health Organization classification guideline for tumours.

### 2.7. Statistical Analysis

This study was descriptive. Therefore, only basic statistical measures such as frequencies, percentages and medians were used to summarise the data. No inferential statistical tests were conducted. Data were summarised and presented as tables.

### 2.8. Consent

A waiver of informed consent was obtained from the Stellenbosch University Health Research Ethics Committee (HREC/REF: S21/07/124).

### 2.9. Ethical Considerations

This study was approved by the Stellenbosch University Health Research Ethics Committee (HREC/REF: S21/07/124). The study was conducted following the South African Good Clinical Practice Guidelines (DOH, 2006) and the Declaration of Helsinki (2013). Data were anonymised using a study number to code each patient folder.

## 3. Results

During the 12-month study period, 113 lesional biopsies were confirmed to be CSCC in 83 patients. Notably, 22 of these patients had multiple CSCCs diagnosed during the study, and this subgroup accounted for 44.2% of the overall number of lesions. The demographics and baseline characteristics of 83 patients meeting the inclusion criteria are presented in Table 1.

The clinical notes lacked consistent documentation of clinical borders and lesion sizes. 15.9% of lesions had poorly defined borders, categorising them as high-risk lesions. Additionally, 8% of CSCCs were larger than 4 cm in diameter, classifying them as “very” high-risk lesions. In most cases (88.5%), the treating clinician had included CSCC in their differential diagnosis on the pathology request form, while 11.5% of lesions were not clinically suspected to be CSCCs.

**Figure 1:**
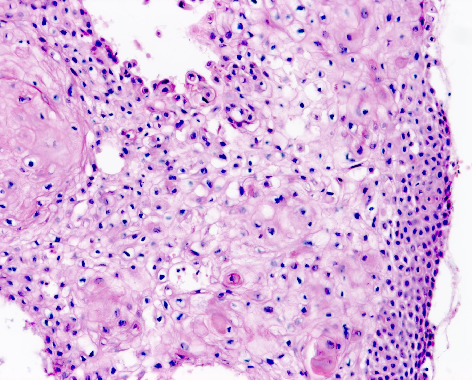
Clear cell squamous cell carcinoma. More than 25% of the tumour cells display clear vacuolated cytoplasm with foci of keratinisation (haematoxylin and eosin staining ×200).

**Figure 2:**
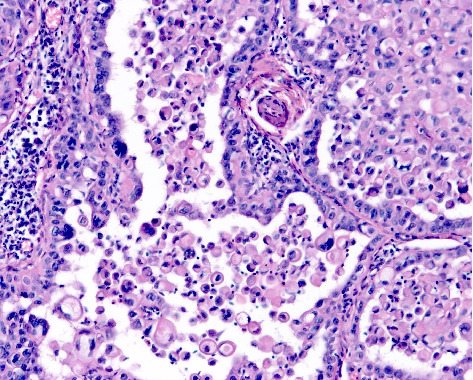
Acantholytic squamous cell carcinoma. Loss of cohesion between individual tumour cells results in variably sized pseudoglandular spaces containing acantholytic tumour cells (haematoxylin and eosin staining ×100).

**Figure 3:**
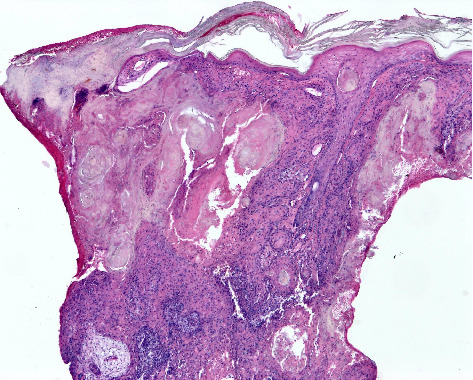
Keratoacanthoma-like squamous cell carcinoma. Well-differentiated invasive aggregates of squamoid tumour cells with infundibular keratinisation and part of an associated keratotic crater with an overhanging epidermal lip (haematoxylin and eosin staining ×40).

**Figure 4:**
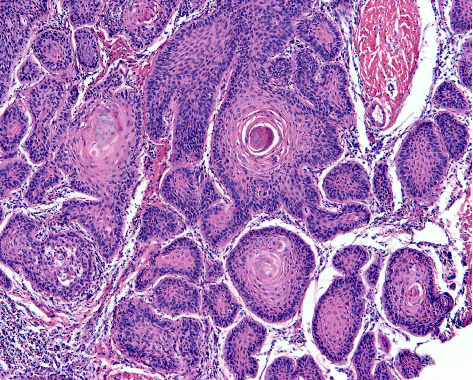
Well-differentiated squamous cell carcinoma. Groups of well-differentiated invasive squamous cell carcinoma display central plugs (pearls) of keratinisation with parakeratosis (haematoxylin and eosin staining ×100).

**Figure 5:**
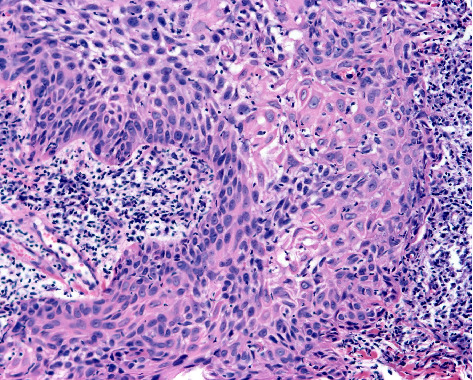
Moderately differentiated squamous cell carcinoma. Infiltrative groups of squamous cell carcinoma are composed of atypical keratinocytes with eosinophilic cytoplasm, individual cell keratinisation and intercellular bridges but without plugs of keratinisation (haematoxylin and eosin staining ×200).

**Figure 6:**
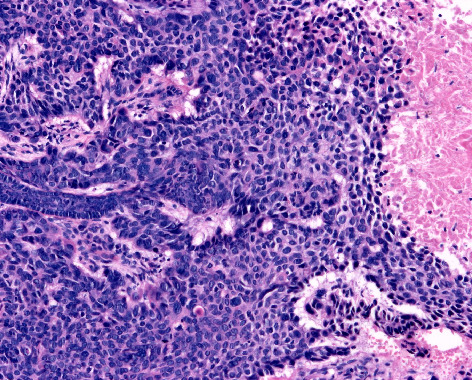
Poorly differentiated squamous cell carcinoma. Infiltrative squamous cell carcinoma groups comprise keratinocytes with pleomorphic, hyperchromatic nuclei, numerous mitoses, and a high nuclear-to-cytoplasmic ratio without discernible keratinisation (haematoxylin and eosin staining ×200).

Histopathological analysis revealed that 13.3% of lesions were keratoacanthoma-like CSCCs (Figure 1), while clear cell and acantholytic subtypes accounted for 6.2% and 5.3%, respectively (Figures 2 and 3). Invasive CSCCs comprised at least 56.6% of cases, with in situ lesions representing 23.9%. Regarding tumour differentiation, 63.7% were well differentiated, 34.5% were moderately differentiated, and 0.9% were poorly differentiated (Figures 4, 5 and 6). Detailed tumour characteristics for all 113 lesions are summarised in Table 2.

None of the lesions exhibited perineural or lymphovascular invasion on histopathological examination. A single patient reported neurologic symptoms suggestive of perineural invasion, but this could not be confirmed on histology. Solar elastosis was present in a significant proportion (57.5%) of lesions, while it was absent in 6.2% of cases. In contrast, only 16.9% of patients had a documented history of chronic exposure to UVR.

By utilising the NCCN risk guidelines, most (77.0%) lesions were classified as high risk, 15.9% as low risk and 7.1% as very high risk. Regarding the very high-risk lesions, seven were clinically larger than 4 cm in size, and one lesion was poorly differentiated on histology. Only a minor proportion (1.8%) of CSCCs (*n* = 2) were identified as recurrences of previously treated lesions. A single lesion arose within an area of chronic inflammation, representing a recalcitrant discoid lupus erythematosus (DLE) plaque on the dorsal hand. None of the lesions arose from regions of prior radiotherapy.

**Figure 7:**
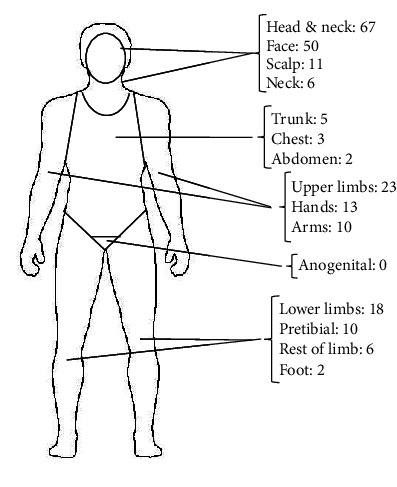
Anatomic distribution of all cutaneous squamous cell carcinomas (*n* = 113).

**Figure 8:**
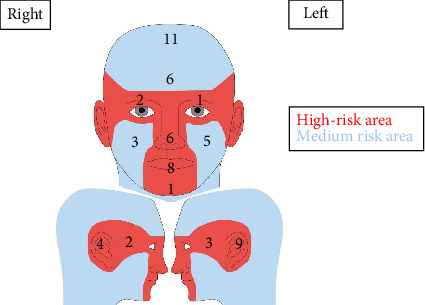
Anatomic distribution of cutaneous squamous cell carcinomas on the face and scalp only (*n* = 61). The area in red represents the high-risk mask area of the face (*n* = 36), while the blue area indicates the rest of the face and scalp, which is considered medium risk (*n* = 25).

The distribution of lesions is illustrated in Figures 7 and 8. CSCCs were more frequently observed on the left side of the face (*n* = 18, 15.9% of all lesions) compared to the right side (*n* = 11, 9.7%). Regarding the face, the lower lip had the highest number of lesions (*n* = 9, 8.0%), followed by the nose (*n* = 6, 5.3%) and forehead (*n* = 6, 5.3%). Other areas with a significant proportion of lesions included the dorsal hands (*n* = 13, 11.5%), the scalp (*n* = 11, 9.7%) and the pretibial area (*n* = 10, 8.8%). The ratio of invasive lesions to in situ lesions on the head was 2.2:1, while this ratio was 3:1 for the hands. The distribution of invasive and in situ lesions was quite similar for the other cutaneous sites.

The predominant method for sampling lesions involved a punch biopsy, which was utilised for 62.8% of the lesions. Cautery and electrodesiccation (C&E) and shave biopsies contributed to the diagnosis of 32.7% and 4.4% of the lesions, respectively. Incisional or excisional biopsies yielded no diagnoses of CSCC during the 12-month study period.

Among all CSCCs diagnosed in the Dermatology OPD, approximately 51.3% were referred to another surgical discipline for definitive resection, with the majority being directed to the Division of Plastic and Reconstructive Surgery. Notably, three cases of lower lip invasive CSCCs were referred simultaneously to the Divisions of Plastic and Reconstructive Surgery and Otorhinolaryngology. Additionally, a single CSCC originating from the fingernail bed was confirmed and referred to the Division of Orthopaedic Surgery for further treatment.

By contrast, around 30.1% of the lesions were managed entirely within the Dermatology OPD without requiring referral to another specialised division. Among these cases, 15 lesions received vigorous cryotherapy with a double freeze–thaw cycle. Thirteen of these lesions were in situ CSCCs; the remaining two were small (less than 2 cm in diameter), invasive, well-differentiated CSCCs in older patients who opted for this specific treatment.

Thirteen lesions were followed up solely with close clinical observation at the Dermatology OPD. Two lesions confirmed to be CSCC by punch biopsy and C&E were later excised with the appropriate surgical margins at the Dermatology OPD. After initial confirmatory biopsies, four in situ lesions received topical therapies, such as imiquimod or 5-fluorouracil. Significantly, 18.6% of the lesions were lost to follow-up. The management of all 113 confirmed CSCCs is detailed in Table 3.

## 4. Discussion

CSCC is the second-most prevalent skin cancer in South Africa and internationally [1, 3]. Compared to BCC, CSCC has an increased potential to exhibit aggressive behaviour and is associated with higher morbidity and mortality [7–10]. The South African population is diverse and exposed to multiple CSCC risk factors [11–17]. Despite this, scientific literature about CSCC in South Africa is scarce, which limits the development of nuanced prevention, detection and management strategies. To address this gap, the present study examined the clinical, histopathological and risk profile characteristics of patients with confirmed CSCC seen over 1 year at a tertiary dermatology unit in the Western Cape Province.

### 4.1. Risk Factors Related to CSCC Development

#### 4.1.1. Skin Pigmentation and Fitzpatrick Phototype

In the present study, most CSCC cases occurred in older males with Fitzpatrick phototype I skin, with only one poorly differentiated lesion observed in a female patient of the same phototype. This finding reaffirms the well-established relationship between reduced skin pigmentation and increased susceptibility to skin cancers induced by UVR exposure [8, 9, 18]. Phototypes II and III comprised 28% of the cohort, while phototypes IV and V accounted for 7.2% each; no cases were recorded among individuals with phototype VI. The median age at diagnosis varied considerably by phototype, with patients of phototype I diagnosed at an average of 73 years, compared to 47 years for phototype IV and 42 years for phototype V. This finding was consistent with the existing literature, which indicates that among South Africans with darker skin tones, CSCC tends to present at a younger age [19–23].

Approximately 81.4% of the South African population is classified as “black” [24], but this group comprises a broad spectrum of Fitzpatrick phototypes [25]. Although CSCC is generally less common in individuals with darker skin phototypes [26], it remains the most frequently diagnosed cutaneous malignancy among “black” South Africans [27]. It also tends to present at a more advanced stage in this population, in part due to persistent misconceptions that darker skin confers immunity against skin cancer [21, 28]. It is also important to note that OCA is highly prevalent in sub-Saharan Africa [17]. OCA is characterised by a markedly reduced or complete lack of skin pigmentation and an elevated risk of early-onset CSCCs that tend to be more aggressive [17]. This was exemplified in the present study by a 57-year-old female with OCA who had a history of multiple invasive CSCCs beginning in adolescence, which was attributed to insufficient photoprotective measures during her childhood.

Public health strategies addressing CSCC in South Africa must be inclusive and reflect the diverse population they serve. Community education, culturally appropriate outreach and early intervention programs can help dispel myths, enhance CSCC prevention efforts and promote earlier detection of CSCC.

#### 4.1.2. Ultraviolet Radiation Exposure

High-dose, intermittent UVR exposure, particularly episodes leading to sunburn in childhood and adolescence, has been associated with an increased risk of developing malignant melanoma and BCC later in life. In contrast, CSCC is more strongly linked to cumulative, long-term sun exposure, and it remains the most significant environmental risk factor for CSCC [8, 9, 29, 30]. In the present study, histological evidence of solar elastosis, a marker of chronic photodamage, was observed in 57.5% of lesions. However, only 19.3% of patients had a documented history of chronic solar UVR exposure. This discrepancy suggests substantial underreporting of UVR exposure in clinical records, highlighting the need for more consistent documentation practices and a standardised, practical system for capturing UVR exposure history at the TAH Dermatology OPD. Implementing structured tools, such as electronic checklists, targeted consultation questions or self-administered patient intake forms in waiting rooms, can improve data accuracy and risk stratification, facilitating counselling and early intervention for high-risk individuals [31].

In addition to environmental exposure, therapeutic UVR plays a role in the development of CSCC [32, 33]. Two patients in the present study developed multiple invasive CSCCs following extensive psoralen and UVA (PUVA) therapy in early adulthood, underscoring the importance of long-term dermatologic surveillance in every patient with a history of PUVA therapy.

#### 4.1.3. Chronic Inflammation

CSCC can develop in nonhealing ulcers or longstanding inflammatory lesions [8, 9]. In the present study, a 47-year-old patient with phototype IV skin developed invasive CSCC within a persistent DLE plaque. The association between CSCC and DLE has been noted in previous South African case series, particularly among younger individuals with darker skin phototypes [34–36]. Patients with DLE should be counselled about their elevated risk of CSCC, primarily when lesions are poorly controlled or recalcitrant to treatment. Regular clinical monitoring, consistent use of antimalarials, such as chloroquine, and strict adherence to sun protection measures may help mitigate this risk [34–37]. Further research is needed to inform preventive and long-term management strategies in South African patients living with DLE.

#### 4.1.4. Immunosuppression

Immunosuppressed individuals, such as those receiving immunomodulatory therapy, people living with HIV and those with chronic lymphocytic leukaemia, are at significantly increased risk of developing CSCC [15, 21, 38–43]. Among organ transplant recipients, the risk is elevated 65–250-fold, often with earlier onset and a more aggressive course [44]. One study found that patients with 10 or more CSCCs most often had underlying immunosuppression [45]. Five patients in the present study had ongoing immunosuppression: Two were HIV-positive, one was on azathioprine for eosinophilic granulomatosis with polyangiitis, and two were receiving methotrexate (one for rheumatoid arthritis, the other for psoriasis). The psoriasis patient had also received PUVA therapy and ciclosporin in early adulthood, a combination strongly linked to increased CSCC risk [33, 46].

South Africa has one of the highest HIV prevalence rates in the world, affecting approximately 19.1% of the population [47, 48]. Despite this, only a few local studies have explored the relationship between HIV and CSCC. A recent study in the Northern Cape Province found an increased incidence of CSCC in younger, darker-skinned individuals, partially attributed to the regional HIV burden [23]. Another case–control study reported a 2.6-fold increase in CSCC risk among HIV-positive patients, with lesions presenting at atypical anatomical sites [16]. Similarly, in the present study, the youngest patients (aged 32 and 42) with CSCC were both HIV-positive with Fitzpatrick phototype V skin. One of the patients presented with a lesion on the fingernail bed, a rare location associated with high-risk HPV subtypes [49–51], which occur more frequently in people living with HIV [41, 52, 53].

These findings underscore the importance of initiating regular skin examinations early in immunosuppressed individuals. Preventive measures, including the use of acitretin in solid organ transplant recipients, should be considered to reduce the risk of CSCC [54]. Clinicians must remain alert to potential malignancies, clearly communicate patients' heightened risk and maintain a low threshold for biopsy of any suspicious lesions.

#### 4.1.5. Cigarette Smoking

In 2017, 19.3% of South African adults reported cigarette use [48]. In contrast, 38.6% of patients in the present study had a history of cigarette smoking. A direct link between smoking and CSCC remains unclear due to conflicting evidence [55–57], although smoking is strongly associated with the development of oral squamous cell carcinoma [58, 59]. This may be relevant to the present study, as seven of the eight lower lip CSCC lesions occurred in current or former smokers. Further research is required to clarify the role of cigarette smoking in CSCC pathogenesis in the South African context, and this should include an exploration of emerging trends, such as vaping and e-cigarette use, and their long-term oncogenic potential.

### 4.2. Shifting BCC-to-CSCC Ratio and Implications for Public Health

The findings of the present study reflect a broader epidemiological trend observed in Australia and the United States: the rising incidence of CSCC relative to BCC [4–6]. In the present study, the BCC-to-CSCC ratio was 1.71:1, a notable decrease from the 2.03:1 ratio recorded at the same institution just 3 years prior [60]. The increasing incidence of CSCC relative to BCC is supported by data from the South African National Cancer Registry, which revealed a decline in the national BCC-to-CSCC ratio from 2.3:1 in 2018 to 1.9:1 in 2023 [27, 61]. Recent data from the Northern Cape Province revealed a higher incidence of CSCC than BCC, overturning the region's expected BCC-to-CSCC ratio [23].

This evolving pattern has significant clinical and public health implications. CSCC is typically more aggressive than BCC and carries a higher risk of recurrence, metastasis and mortality [7–10]. Consequently, a rise in CSCC cases may translate into a disproportionate increase in resource utilisation and treatment complexity. It could place further strain on the South African public healthcare system, given that a substantial proportion of the country's healthcare expenditure is already directed towards diagnosing and managing nonmelanoma skin cancers [62].

### 4.3. Lesion Location and Anatomical Risk

The anatomical site plays a crucial role in determining the prognosis and management of CSCC [63]. In the present study, most lesions were on the head and neck, followed by the dorsal hands and pretibial areas, which are all sites characterised by high cumulative UVR exposure and an elevated risk of recurrence [63]. These regions also demonstrated a higher proportion of invasive CSCCs relative to in situ lesions, reinforcing the role of photodamage in tumour development. Notably, 19% of all lesions were located on the ear and lower lip; lesions in these areas have an increased metastatic risk compared to lesions on other sun-exposed sites [7, 64, 65]. Interestingly, more CSCCs were diagnosed on the left side of the face than on the right in the present study; a similar pattern was previously observed in a cohort of patients with BCC at the same institution [66]. The reason for this left-sided predominance remains unclear and warrants further investigation.

### 4.4. Biopsy Techniques and Their Clinical Implications

Accurate CSCC risk stratification depends significantly on the biopsy technique used [63]. The present study sampled most lesions via punch and superficial shave biopsy, or C&E. For patients in high-risk groups who frequently develop multiple lesions, destructive modalities like C&E are often favoured for clinically low-risk tumours, enabling treatment of several lesions in a single visit [63]. While C&E is time-efficient and cost-effective, with both diagnostic and therapeutic potential, it may not reliably assess tumour depth, margin status or histological features such as perineural invasion or solar elastosis [63]. When C&E is performed based solely on clinical judgement, histopathological review is essential to rule out high-risk features that may warrant further or more aggressive intervention [63].

A relatively high number of patients in the present study who underwent C&E failed to return for follow-up. A possible explanation for this was that patients mistakenly believed that the procedure was completely curative. It is essential to have clear communication between clinicians and patients regarding the limitations of each method of tumour sampling and the necessity of histopathological review at follow-up. Clinicians must carefully weigh the diagnostic limitations of the biopsy technique when deciding which to utilise.

### 4.5. Tumour Depth as a Prognostic Indicator

Tumour thickness remains a reliable prognostic marker in CSCC. Lesions with greater depth are associated with an increased risk of local recurrence and metastasis [63–65, 67]. The NCCN guidelines classify CSCCs with a depth of 6 mm or greater as “very high risk,” a threshold supported by other studies [63, 64, 67]. Encouragingly, none of the lesions in the present study exceeded 6 mm in thickness. In situ CSCCs, defined by full-thickness epidermal atypia without dermal invasion, accounted for nearly a quarter (23.9%) of cases [68]. These lesions were primarily managed with nonsurgical modalities such as cryotherapy, topical 5-fluorouracil or imiquimod, with only a minority referred for surgical excision. This treatment approach aligns with NCCN recommendations, which endorse nonsurgical therapies for focal in situ lesions, particularly those arising within actinic keratoses [63].

### 4.6. Histological Subtypes

Accurate identification of the histological subtype of CSCC is crucial for determining prognosis and informing treatment decisions and follow-up strategies [7, 69]. Specific histological variants, including the acantholytic, adenosquamous, metaplastic and spindle cell variants, are associated with a higher risk of local recurrence and generally require more aggressive management [63, 69]. Desmoplastic CSCC, in particular, is categorised by the NCCN as a “very high-risk” subtype due to its pronounced tendency for recurrence and metastasis [63, 64, 67]. In the present study, most lesions (75.2%) were of the conventional subtype, as expected. Acantholytic CSCCs represented 5.3% and were exclusively located on the face, aligning with an area of high cumulative UVR exposure. Clear cell CSCCs comprised 6.2% of lesions and lacked a consistent anatomical distribution; while their clinical implications remain poorly defined, they are currently regarded as carrying an indeterminate risk [68].

Lesions diagnosed as keratoacanthomas (KAs) were deliberately excluded from the study. Although KAs often resemble well-differentiated CSCCs clinically and histologically, their biological nature remains a matter of contention [70–73]. The absence of universally accepted criteria for distinguishing KA from well-differentiated CSCC underscores dermatopathological diagnostic challenges. However, recent advances in morphological, molecular and immunological profiling increasingly support the view that KAs represent a distinct but related benign entity [70–73]. This reinforces the importance of nuanced histological evaluation of cutaneous tumours.

### 4.7. NCCN Risk Stratification of Lesions

Risk stratification plays a critical role in managing CSCC, as specific subgroups of lesions are associated with higher risks of recurrence, metastasis and mortality [63]. The NCCN guidelines provide a validated framework for stratifying lesions into low, high and very high-risk categories [63, 74], and recent studies have confirmed their utility in predicting adverse clinical outcomes [74]. In the present study, 15.9% of lesions were classified as low risk, 77.0% as high risk and 7.1% as very high risk. However, these proportions may have underestimated the full burden of aggressive disease due to inconsistent documentation of key clinical parameters required for accurate risk categorisation, namely, lesion size and border definition. This limitation underscores the importance of adopting structured, evidence-based frameworks, such as the NCCN guidelines, to enhance the completeness and consistency of clinical note-taking, thereby supporting more accurate risk assessment.

### 4.8. Clinical Implications of Multiple Primary Lesions

A substantial proportion of patients (26.5%) in the present study had been diagnosed with more than one new CSCC within 12 months, which is clinically relevant, as having multiple lesions is associated with a heightened risk of local recurrence and lymph node metastasis [45]. As such, it is essential to thoroughly document the number and specific locations of previous CSCCs in patients with multiple lesions. At every consultation, a detailed examination of all scar sites from previously treated CSCCs, along with careful palpation of the corresponding lymph node basins, is recommended.

### 4.9. Management of Lesions

The majority of high-risk and very high-risk CSCCs in this study were appropriately referred to surgical specialities at TAH, underscoring the critical role of multidisciplinary care in managing aggressive skin cancers. Surgical intervention remains the cornerstone of treatment for high-risk lesions due to the increased likelihood of local recurrence and metastasis [7, 10, 75]. Among the eight lesions classified as very high risk in the present study, seven were managed surgically by the Plastic and Reconstructive Surgery or ENT Divisions; referral was not pursued in one case due to the patient's comorbidities. This large (6 cm) in situ lesion on the pretibial area of an 82-year-old patient was successfully managed nonsurgically with topical 5-fluorouracil. This case emphasises individualising treatment plans and highlights the real-world complexities that limit the implementation of ideal clinical pathways.

## 5. Study Limitations

This retrospective study is subject to several limitations that may affect the reliability and generalisability of the findings. First, the analysis relied on the completeness and accuracy of clinical records, which may introduce information bias. The study cohort was limited to patients referred to the TAH Dermatology OPD, potentially excluding cases from the broader referral area and introducing selection bias, particularly if lesions in anatomically sensitive regions (e.g., anogenital sites) were managed elsewhere. Additionally, skin phototypes were classified using the Fitzpatrick scale. This widely adopted but inherently subjective tool may inadequately capture the diversity of pigmentation and UVR responses in South African populations. The onset of the COVID-19 pandemic further impacted the study, leading to reduced clinic attendance during the latter part of the study period and loss of follow-up in some cases.

## 6. Conclusion

The South African population is faced with significant risk factors related to CSCC. Despite this, there is a notable lack of scientific literature characterising CSCC within the local context. The present study aimed to address this gap by examining the clinical, histopathological and risk profiles of CSCC in a South African cohort at a tertiary dermatology unit in the Western Cape Province. This study provides valuable insights into the heterogeneous nature of CSCC in South Africa and the associated challenges. Prospective research across diverse settings will be key to improving CSCC prevention, early detection and management strategies nationally.

## Figures and Tables

**Table 1:** Patient demographic and baseline characteristics (*n* = 83).

Variable	Frequency (*n*)	Percentage (%)
Gender		
Male	50	60.2
Female	33	39.8
Fitzpatrick skin phototype		
I	54	65.1
II	16	19.3
III	7	8.4
IV	3	3.6
V	3	3.6
VI	0	0.0
UV exposure		
Chronic sun exposure	14	16.9
Psoralen and ultraviolet A	2	2.4
UV exposure history unknown	67	80.7
Other risk factors		
Discoid lupus erythematosus	1	1.2
Oculocutaneous albinism	1	1.2
Immunosuppression		
HIV infection	2	2.4
On immunosuppressive therapy	3	3.6
History of cigarette smoking		
No	37	44.6
Yes	32	38.6
Not documented	14	16.9

**Table 2:** Clinical and histopathological data of all cutaneous squamous cell carcinomas (*n* = 113 biopsy samples).

Variable	Frequency (*n*)	Percentage (%)
Clinical diameter		
< 2 cm	46	40.7
2–4 cm	26	23.0
≥ 4 cm	9	8.0
Not documented	32	28.3
Borders		
Well defined	33	29.2
Poorly defined	18	15.9
Not documented	62	54.9
Diagnostic procedure		
Punch biopsy	71	62.8
Cautery and electrodesiccation	37	32.7
Shave biopsy	5	4.4
Incisional biopsy	0	0.0
Excision ellipse biopsy	0	0.0
Degree of differentiation		
Well-differentiated	72	63.7
Moderately differentiated	40	34.5
Poorly differentiated	1	0.9
Histologic subtype		
None	85	75.2
Keratoacanthoma-like	15	13.3
Clear cell	7	6.2
Acantholytic	6	5.3
Depth (thickness or level of invasion)		
CSCC in situ	27	23.9
≤ 6 mm and no invasion beyond subcutaneous fat	18	15.9
> 6 mm or invasion beyond subcutaneous fat	0	0.0
At least CSCC in situ	11	9.7
Invasive; unable to assess the extent of invasion	46	40.7
Unable to assess due to the nature of the biopsy	11	9.7
Solar elastosis		
Present	65	57.5
Absent	7	6.2
Unable to assess	41	36.3
NCCN risk stratification		
Low risk	18	15.9
High risk	87	77.0
Very high risk	8	7.1
High-risk features on history and physical examination		
Head, neck, hands, feet, pretibial and anogenital (size < 4 cm)	92	81.4
Trunk, extremities (with size 2–< 4 cm)	26	23.0
Poorly defined clinical borders	18	15.9
Rapidly growing tumour	14	12.4
Background of immunosuppression	5	4.4
Recurrent lesion	2	1.8
Site of chronic inflammatory process	1	0.9
Neurologic symptoms	1	0.9
High-risk features on histology		
Acantholytic subtype	6	5.3

**Table 3:** Management of all confirmed CSCCs (*n* = 113).

Variable	Frequency (*n*)	Percentage (%)
Plastic surgery referral	47	41.6
Lost to follow-up	21	18.6
Vigorous cryotherapy	15	13.3
Close follow-up at the Dermatology OPD only	13	11.5
Otorhinolaryngology referral	7	6.2
Plastic surgery plus otorhinolaryngology referral	3	2.7
Topical 5-fluorouracil	3	2.7
Elliptical excision in the Dermatology OPD	2	1.8
Orthopaedic surgery referral	1	0.9
Topical imiquimod	1	0.9
Cautery and electrodesiccation	0	0.0

## Data Availability

Data can be available on request. The requesting party can contact the corresponding author.
